# Ceramide d18:1/24:1 as a potential biomarker to differentiate obesity subtypes with unfavorable health outcomes

**DOI:** 10.1186/s12944-023-01921-0

**Published:** 2023-10-04

**Authors:** Baowen Yu, Moran Hu, Wanzi Jiang, Yizhe Ma, Jingya Ye, Qinyi Wu, Wen Guo, Yan Sun, Min Zhou, Yiwen Xu, Zhoulu Wu, Yiwen Wang, Sin Man Lam, Guanghou Shui, Jingyu Gu, John Zhong Li, Zhenzhen Fu, Yingyun Gong, Hongwen Zhou

**Affiliations:** 1 Department of Endocrinology, the First Affiliated Hospital of Nanjing Medical University, Nanjing, Jiangsu China; 2 Institute of Genetics and Developmental Biology, Chinese Academy of Sciences, Beijing, China; 3 Department of Biochemistry and Molecular Biology, Nanjing Medical University, Nanjing, Jiangsu China

**Keywords:** Metabolically unhealthy obesity, Ceramide, Cer d18:1/24:1, Cardiovascular disease

## Abstract

**Background:**

The criteria for metabolically healthy obesity (MHO) and metabolically unhealthy obesity (MUO) remain controversial. This research aimed to identify a potential biomarker to differentiate the subtypes of obesity.

**Methods:**

The study conducted a lipidomic evaluation of ceramide in the serum of 77 Chinese adults who had undergone hyperinsulinemic-euglycemic clamps. These adults were divided into three groups according to the clinical data: normal weight control group (N = 21), MHO (N = 20), and MUO (N = 36).

**Results:**

The serum Cer d18:1/24:1 level in the MHO group was lower than that in the MUO group. As the Cer d18:1/24:1 level increased, insulin sensitivity decreased, and the unfavorable parameters increased in parallel. Multivariate logistic regression analysis revealed that serum Cer d18:1/24:1 levels were independently correlated with MUO in obesity. Individuals with higher levels of Cer d18:1/24:1 also had an elevated risk of cardiovascular disease. Most ceramide subtype levels increased in obesity compared to normal-weight individuals, but the levels of serum Cer d18:0/18:0 and Cer d18:1/16:0 decreased in obesity.

**Conclusions:**

The relationships between ceramide subtypes and metabolic profiles might be heterogeneous in populations with different body weights. Cer d18:1/24:1 could be a biomarker that can be used to differentiate MUO from MHO, and to better predict who will develop unfavorable health outcomes among obese individuals.

**Trial registration:**

The First Affiliated Hospital of Nanjing Medical University’s Institutional Review Board authorized this study protocol, and all participants provided written informed consent (2014-SR-003) prior to study entry.

**Supplementary Information:**

The online version contains supplementary material available at 10.1186/s12944-023-01921-0.

## Background

Obesity now represents a worldwide public health problem, impacting the health of a significant number of people worldwide. 107.7 million children and 603.7 million adults were identified as obese in 2015 according to the Global Burden of Disease Obesity Collaborators. Approximately 4.0 million deaths, mostly due to cardiovascular disease (CVD), are related to high body mass index (BMI) [1]. These findings indicated the importance of effective treatments for decreasing obesity prevalence and disease burdens.

Obesity is associated with many kinds of metabolic abnormalities and diseases, including type 2 diabetes mellitus (T2DM), nonalcoholic fatty liver disease (NAFLD), and CVD [2]. Obese individuals who exhibit fewer metabolic dysfunctions are considered metabolically healthy obesity (MHO), and they may represent a unique group or a group that is in the process of transitioning to metabolically unhealthy obesity (MUO) [3]. In contrast, MUO was reported as a less favorable type of obesity with impaired lipid and glucose metabolism and a high risk of cardiovascular and inflammatory abnormalities and other metabolic disorders [4]. There are many definitions for MUO, most of which are based on phenotypes with unfavorable laboratory findings, such as inflammatory markers, metabolic parameters, insulin sensitivity, fibrinolytic activity, and liver function [2]. Some researchers also believed that the different risks of cardiometabolic diseases are the main identified factors for MHO and MUO [5]. However, the various definitions of MHO and MUO are still controversial [3]. The current classifications lack standardization and may not be sufficient or accurate to identify the specific obese subgroup. There is thus a need to have a better biological marker that can make the classification of obesity more accurate, with better prediction of health outcomes so that interventions can be initiated earlier to improve the disease outcomes.

Sphingolipids (SLs), the minor components of membranes, have crucial biological functions such as altering the physiochemical characteristics of lipid bilayers and influencing the activation of intracellular proteins and receptors. Although SLs comprise only 2–15% of the total cellular lipidome, they play important roles in the development of metabolic diseases and CVD [6]. Ceramide, one of the best characterized SLs, represents a heterogeneous group of lipids that are identified by the specific fatty acyl moiety bonded to sphingosine with an amide bond. The different fatty acyl moieties encompass short to long fatty acids (C2-C34) [7]. Ceramides, acting as secondary messengers for cellular signaling, are related to both lipid and glucose metabolism [8]. As reported before, ceramides were proven to play crucial roles in cell proliferation, autophagy, apoptosis, senescence, migration, regulating mitochondrial dynamics, lipid utilization, glucose sensitivity and inflammation [9–11]and were associated with depression [12], cancer [13], and neurodegenerative disorders [14]. However, in recent years, ceramides are getting increased attention for their important roles in metabolic dysfunctions such as obesity, hepatic steatosis, diabetes, and CVD [15]. Although increased levels of ceramides were shown to be associated with obesity [16], no study has assessed ceramides levels with regard to MHO or MUO. This study measured the ceramide levels in both MHO and MUO to evaluate the efficiency of ceramides in differentiating the subtypes of obesity with unfavorable health outcomes.

## Methods

### Data source

Data were derived from a cohort of participants who had undergone both hyperinsulinemic-euglycemic clamp and lipidomic analysis in the First Affiliated Hospital of Nanjing Medical University, Nanjing, China [17]. The participants who underwent the hyperinsulinemic-euglycemic clamp had not taken any medication or supplement, had no history of smoking or high alcohol intake (four or more standard drinks per week for men and two or more standard drinks per week for women), severe disease, acute inflammation or pregnancy. Individuals with blood samples of poor quality and missing physical examination data were excluded when the lipidomic analysis was conducted.

### Study population

The eligibility criteria for the study included normal weight or obese individuals aged 18–55 years old. Obesity and normal weight were defined as a BMI ≥ 28 kg/m2 and 18.5–23.9 kg/m2 respectively based on Chinese standards [18]. We excluded subjects whose ceramide subtype concentrations were below the limit of detection. A total of 77 individuals were finally enrolled. Demographic characteristics and physical examination data included sex, age, geographic region, body mass index (BMI), systolic/diastolic blood pressure (SBP/DBP) and waist circumference (WC). These individuals were divided into three groups based on their BMI and metabolic parameters: normal weight control (NC) group, MHO and MUO. None of the individuals in the NC group had evidence of hypertension, hyperlipidemia, diabetes, or any other diseases. Obese individuals who met at least three of the following criteria were defined as MUO based on metabolic syndrome: (1) 1. WC ≥ 90 cm in men or ≥ 80 cm in women (recommended by WHO for WC Threshold for abdominal obesity in Asian); (2) Fasting serum triglycerides ≥ 150 mg/dL (1.7 mmol/L); (3) HDL-C ≤ 40 mg/dL (1.0 mmol/L) in men or ≤ 50 mg/dL (1.3 mmol/L) in women; (4) SBP ≥ 130 mmHg and/or DBP ≥ 85 mmHg; and 5.Fasting glucose ≥ 100 mg/dL or diagnosed with diabetes [19]. The other individuals were classified as MHO.

### Hyperinsulinemic-euglycemic clamp

The hyperinsulinemic-euglycemic clamp method established by DeFronzo et al. was used to assess whole-body insulin sensitivity [20]. The specific clamp test details were described previously [17]. The hepatic glucose production could be suppressed by a high dose of insulin use (> 80 mU/m2*min). When the circulation blood glucose is stable, the rate of glucose infusion (GIR) is equals to that of whole-body glucose disposal (GDR) representing body insulin sensitivity. After a preparation of persistent insulin infusion, the body can achieve a steady-state insulin concentration. The glucose infusion ensures the plasma glucose concentration at approximately 5 mmol/L. The mean glucose infusion rate for the last 30 min was calculated as GIR_30_ representing body insulin sensitivity.

### Laboratory measurements

The assessments included plasma glucose (using a blood glucose biochemical analyzer -Germany, Biosen). Standard enzymatic assays were used to measure total cholesterol (TC), triglyceride (TG), low-density lipoprotein cholesterol (LDL-c), high-density lipoprotein cholesterol (HDL-c) and other biochemical phenotypes in the laboratory of the First Affiliated Hospital of Nanjing Medical University, Nanjing, China.

### Measurements of ceramide levels

The ceramide data were acquired from a cohort we have reported before. The participants of this cohort had conducted targeted lipidomics of 90 lipid species in 7 classes of lipids, including free fatty acids, sphingomyelins, ceramides, glu-ceramides, lac-ceramides, ganglioside and globotriaosylceramides [17]. Lipids were acquired from serum (20 µL) and dried in a SpeedVac of OH mode according to a modified version of Bligh and Dyer’s extraction method (double rounds of extraction). Lipid extracts were redissolved in a 1:1 (v/v) solution of chloroform: methanol spiked with relevant internal standards before analyzing. The lipidomic analyses were conducted on an Exion UPLC system coupled with a QTRAP 6500 PLUS system (Sciex). Sphingolipids were separated on a Phenomenex Luna Silica 3 μm column (i.d. 150 × 2.0 mm). The chromatographic conditions are as follows: mobile phase A (chloroform : methanol : ammonium hydroxide, 89.5 : 10 : 0.5) and mobile phase B (chloroform : methanol : ammonium hydroxide : water, 55 : 39: 0.5 : 5.5) at a flow rate of 270 µL/min and column oven temperature of 25 °C. Individual sphingolipid species were quantified by reference to spiked internal standards including Cer d18:1/17:0, GluCer d18:1/8:0, LacCer d18:1/8:0, and SM d18:1/12:0, obtained from Avanti Polar Lipids; d3-GM3 d18:1/18:0 and Gb3 d18:1/17:0 purchased from Matreya LLC; d8-FFA 20:4 from Cayman Chemicals; and d31-FFA16:0 from Sigma Aldrich. This study would focus on ceramides and the obesity subtypes, the correlations between other lipids and metabolic disorders had been summarized before [17].

### Risk assessment models

The risk of arteriosclerotic cardiovascular disease (ASCVD) was assessed by the prediction model of China-PAR. The total study sample size of the China-PAR project was more than 127 thousand and the longest follow-up time was more than 23 years. This effective tool has already been proven to have good performance in predicting ASCVD risk in the Chinese population [21, 22]. The risks of individuals were assessed using the following website: http://www.cvdrisk.com.cn (10-year ASCVD risk: high risk ≥ 10. 0%, medium risk: 5.0%~9.9%, low risk < 5.0%). Lifetime ASCVD risk was defined as follows: the risk of developing ASCVD from now to 85 years old, with the cutoff for low and high risk set to 32.8%.) [21, 22]. The ceramide test score (CERT1) (calculated based on the levels of Cer d18:1/16:0, Cer d18:1/18:0, Cer d18:1/24:1, Cer d18:1/24:0) was also calculated in this study to predict the risk of CVDs [23, 24]. CERT1 could classify individuals into low, moderate, increased and high risk of CVDs according to the previous studies [23, 24]. Fibrosis-4 (FIB-4) and nonalcoholic fatty liver disease score (NFS) were used to assess hepatic fibrosis. FIB-4= [age (years) × AST(U/L)]/[PLT (×109/L) ×$\surd$ALT (U/L)]. The cutoff values for stage 0–2 hepatic fibrosis and significant fibrosis are < 1.3 and > 2.67 respectively [25, 26]. NFS = − 1.675 + 0.037 × age (y) + 0.094 × BMI (kg/m^2^) + 1.13 × impaired fasting glucose or diabetes (yes = 1, no = 0) + 0.99 × AST/ALT ratio − 0.013 × platelet count (×109/L) − 0.66 × albumin (g/dL). The presence of advanced fibrosis was detected with good accuracy using the high cut-off point of NFS (0.676), while it was excluded using the low cut-off point (-1.455) of NFS [27].

### Statistics

Continuous variables are represented as the mean ± standard deviation (SD) or median (quartile 1, quartile 3), and categorical variables are represented as the frequency. Individuals with values of variables above or below the mean ± 5 SD were regarded as outliers, and these individuals were excluded. The Mann–Whitney U test was used for data that were non-normally distributed, while Student’s t test was used for data that were normally distributed when the differences between two groups were analyzed. The Kruskal‒Wallis test was used for data that were non-normally distributed, while one-way ANOVA was used for data normally distributed when multiple group differences were analyzed. Categorical variables were compared by the chi-square test. The relationships between ceramides and metabolic characteristics were analyzed by Pearson correlation (normally distributed data) or Spearman correlation (non-normally distributed data). Models of binary logistic regression were fitted to estimate the associations between ceramides and the presence of MUO. The software IBM SPSS 22.0 and statistical software R 4.1.0 were used to conduct the statistical analysis. Statistical significance was defined as P < 0.05.

### Study approval

This study approval was granted by the Institutional Review Board of the First Affiliated Hospital of Nanjing Medical University, and all participants provided written informed consent (2014-SR-003) prior to study entry.

## Results

### Clinical characteristics of the subjects

Among the 77 individuals with BMI 18.73-23.80 kg/m^2^ (normal weight) and 28.01-61.91 kg/m^2^ (obese), 21 (27%) were classified as the NC group, 20 (26%) were classified as MHO and another 36 (47%) were classified as MUO. Basic clinical characteristics based on metabolic phenotypes are displayed in Table 1(the proposed normal ranges of values given by the laboratory for blood parameters are displayed in Supplementary Table 1).

**Table 1:** Clinical characteristics of individuals among the three groups

	NW	OB		
	(mean ± SD)	(mean ± SD)		
	NC (n = 21)	MHO (n = 20)	MUO (n = 36)	P
**Sex (Male : Female)**	10:11	12:8	13:23	0.22^1^
**Age (y)**	25.62 ± 3.78	26.05 ± 8.81	31.03 ± 8.52^b^	0.007^2^
**BMI (kg/m** ^**2**^ **)**	20.75 ± 1.43	36.66 ± 5.27^a^	37.34 ± 6.79^a^	＜0.001^2^
**SBP (mmHg)**	115 ± 7	130 ± 11^a^	144 ± 19^a^	＜0.001^2^
**DBP (mmHg)**	68 ± 7	83 ± 9^a^	91 ± 13^a^	＜0.001^2^
**WC (cm)**	73.78 ± 7.72	110.90 ± 13.84^a^	109.58 ± 11.86^a^	＜0.001^3^
**ALT (U/L)**	18.26 ± 15.43	67.72 ± 55.94^a^	70.02 ± 46.34^a^	＜0.001^2^
**AST (U/L)**	21.94 ± 9.76	45.69 ± 32.18^a^	45.55 ± 29.43^a^	0.001^2^
**TG (mg/dL)**	68.05 ± 22.69	88.16 ± 28.11	202.72 ± 110.35^ab^	＜0.001^2^
**TC (mg/dL)**	157.14 ± 23.60	183.89 ± 37.11^a^	200.23 ± 38.47^a^	＜0.001^3^
**LDL-c (mg/dL)**	87.39 ± 20.40	116.73 ± 32.37^a^	133.13 ± 29.75^a^	＜0.001^3^
**HDL-c (mg/dL)**	57.73 ± 12.54	46.72 ± 8.29^a^	40.82 ± 6.84^a^	＜0.001^2^
**Lp(a) (mg/L)**	186.90 ± 189.80	201.35 ± 185.31	147.68 ± 170.52	0.513^2^
**GIR**_**30**_	10.92 ± 1.84	4.95 ± 1.54^a^	4.10 ± 1.46^a^	＜0.001^3^
**FPG (mg/dL)**	89.13 ± 9.21	86.81 ± 11.41	115.46 ± 32.17^ab^	＜0.001^2^
**SCr (µmol/L)**	69.03 ± 16.56	65.49 ± 12.12	63.89 ± 13.24	0.41^3^
**BUN(mmol/L)**	4.79 ± 1.28	4.46 ± 0.91	4.70 ± 1.15	0.626^3^
**UA (µmol/L)**	348.99 ± 78.48	428.50 ± 119.09^a^	410.93 ± 89.65	0.019^3^

**Abbreviations**: NW: normal weight; OB: obesity; BMI: body mass index; SBP: systolic blood pressure; DBP: diastolic blood pressure; WC: waist circumference; ALT: alanine aminotransferase; AST: aspartate aminotransferase; TG: triglyceride; TC: total cholesterol; LDL-c: low-Density Lipoprotein cholesterol; HDL-c: high-Density Lipoprotein cholesterol; Lp(a): lipoprotein(a); FPG: fasting plasma glucose; SCr: serum creatinine; BUN: blood urea nitrogen; UA: uric acid

^**1**^ The categorical variables among three groups were compared via the chi-square test; ^**2**^The continuous variables among three groups that were not normally distributed or normally distributed variables with heterogeneity of variance were compared via nonparametric tests and with post hoc tests of Kruskal‒Wallis tests. ^**3**^ The continuous variables among the three groups that were normally distributed were compared via one-way ANOVA with Bonferroni correction

^**a**^ Significant differences (P < 0.05) were shown with the NC group through post hoc tests. ^b^ Significant differences (P < 0.05) were shown with MHO through post hoc tests

There was no statistical difference in BMI between the MHO and MUO groups. The distributions of sexes were not significantly different among the three groups. GIR_30_ (which represents whole body insulin sensitivity. see methods.) tended to decrease in MHO/MUO groups compared to the NC group. However, GIR_30_ showed no significant difference between MHO and MUO. LDL-c, the biomarker to assess CVD risk, increased significantly in both MHO and MUO. In general, all the metabolic parameters in the obesity group were worse than those in the NC group.

### Ceramides among the three groups

**Fig. 1:**
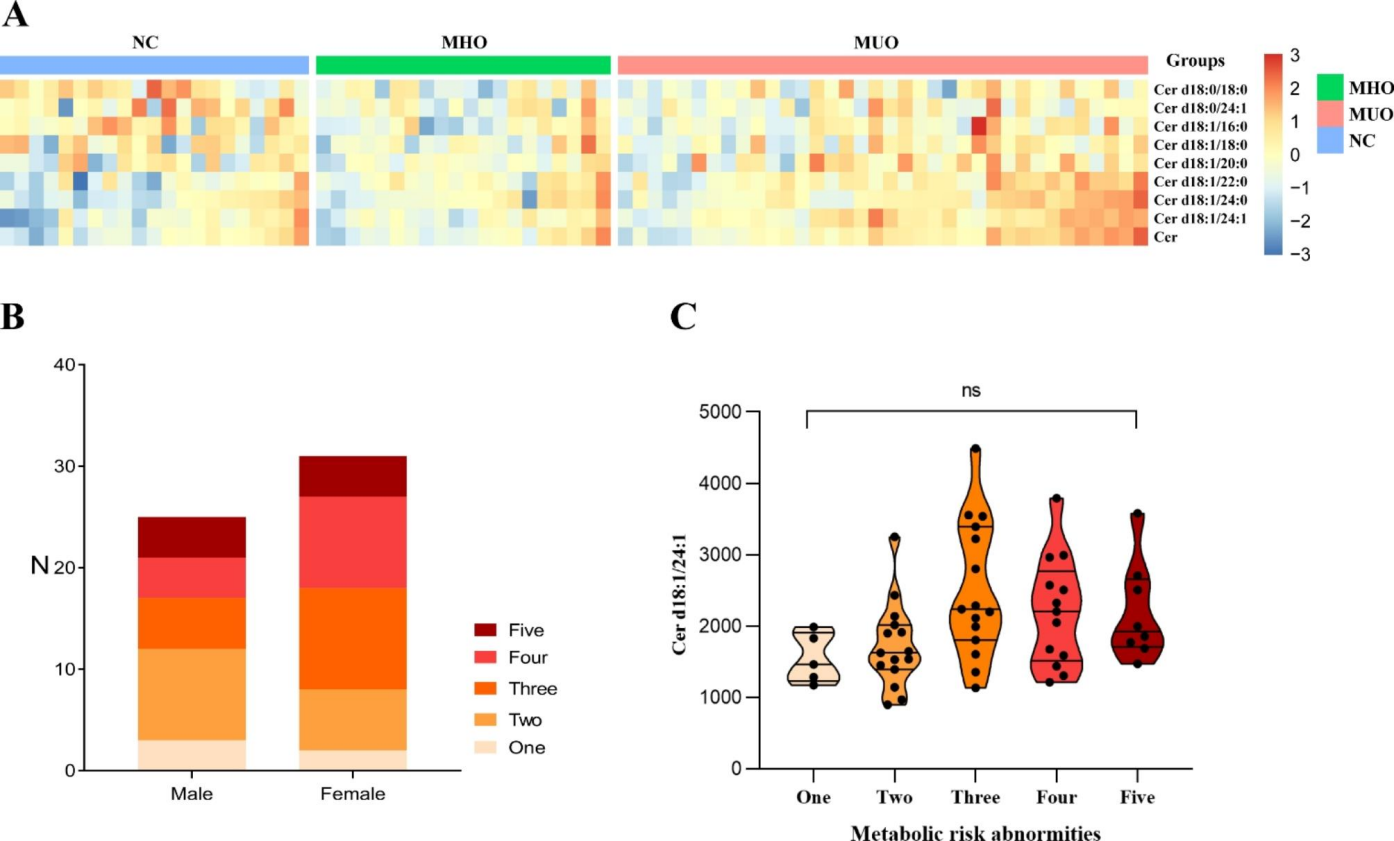
Ceramide distributions in the cohort **A**: The heatmap shows the relative abundances of ceramide levels among the three groups. The concentration values of ceramides were normalized by the logarithm to base 2 and the legend shows the relative abundances in Z score. The color intensity (from blue to red) suggests low to high concentration values. Three groups were distinguished by different colors of upper legends (blue for “NC”, green for “MHO” and pink for “MUO”). **B**: Population distribution of metabolic risk abnormities by sex **C**: The variation tendency of serum Cer d18:1/24:1 levels in populations with “one” to “five” metabolic risk abnormities. The violin plot shows the median and quartiles of ceramide levels, of which the features also show the distribution of data

The concentrations of ceramides were logarithmically transformed into a normal distribution. The differences in ceramide levels among the three groups are shown in Table 2. The heatmap of ceramides (Fig. 1A) visualizes the concentrations. The level of Cer d18:1/24:1 was lower in both the NC and MHO groups than in the MUO group. Total serum ceramide showed a similar trend to Cer d18:1/24:1. Most notably, there were significant increases in serum Cer d18:0/18:0 and Cer d18:1/16:0 levels in the NC group compared with the MHO group. We also compared the levels of Cer d18:0/18:0 and Cer d18:1/16:0 between normal weight and obese individuals. Our results showed that the levels of Cer d18:0/18:0 and Cer d18:1/16:0 were significantly lower in people with obesity.

**Table 2:** Serum Ceramides (nmol/L) of individuals among the three groups

	NW	OB		
	NC (n = 21)	MHO (n = 20)	MUO (n = 36)	P
**Cer d18:0/18:0**	245.1 (132.4,349.6)	105.2 (90.3,217.9)^a^	152.8 (108.7,265.3)	**0.015**
**Cer d18:0/24:1**	165.9 (116.8,352.7)	135.5 (87.5,225.4)	131.4 (95.0,223.0)	0.308
**Cer d18:1/16:0**	740.9 (649.9,994.9)	559.2 (462.5,710.3)^a^	616.1 (545.4,829.2)	**0.028**
**Cer d18:1/18:0**	451.4 (223.4,559.3)	284.8 (232.1,380.0)	328.6 (233.8,458.3)	0.531
**Cer d18:1/20:0**	247.0 (114.6,394.4)	209.4 (158.5,250.3)	250.7 (158.6,452.0)	0.25
**Cer d18:1/22:0**	785.5 (519.9,981.2)	895.6 (753.0,1240.0)	1147.5 (824.3,1464.1)^a^	**0.003**
**Cer d18:1/24:1**	1600.1 (1013.9,2078.8)	1582.3 (1310.7,1969.0)	2201.4 (1676.2,2923.4)^ab^	＜**0.001**
**Cer d18:1/24:0**	2123.8 (1700.9,3391.0)	2680.4 (2048.0,3895.3)	3287.5 (2396.1,4505.9)^a^	**0.025**
**Cer**	7181.1 (5121.9,7926.6)	6326.1 (5563.2,8312.3)	8271.9 (6784.7,10703.3)^ab^	**0.009**

The concentration values of ceramides were transformed into a normal distribution by logarithm

The concentrations of ceramides among the three groups were compared via one-way ANOVA with Bonferroni correction

Continuous variables that were not normally distributed were presented as median (quartile1, quartile3)

^**a**^ Significant differences (P < 0.05) were shown with the NC group through post hoc tests. ^b^ Significant differences (P < 0.05) were shown with MHO through post hoc tests

The obese individuals were then divided into 5 groups on the basis of the numbers of metabolic risk abnormalities (Method: five criteria of metabolic syndrome) (Fig. 1B). The percentage of individuals with two to four metabolic abnormalities was 76.8%. There is no significance different distribution of genders among 5 groups (*P* = 0.435). The level of Cer d18:1/24:1 showed an increasing trend with the accumulation of abnormality numbers (Fig. 1C).

### Correlations between ceramides and multiple clinical characteristics

**Fig. 2:**
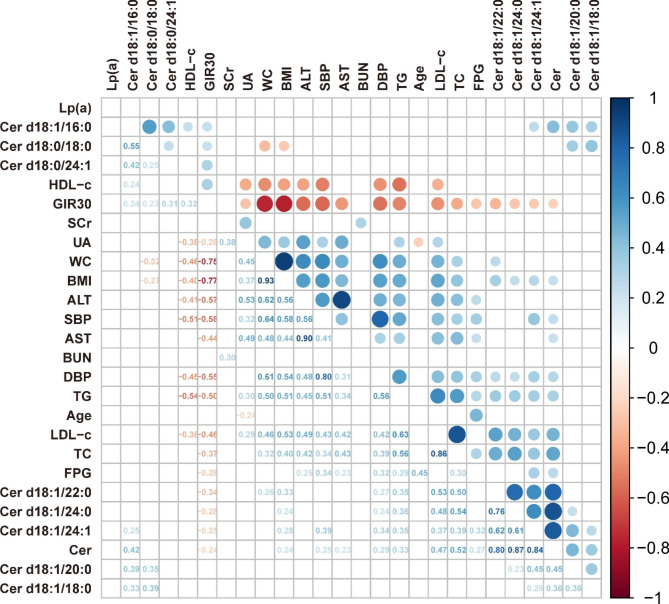
Correlations between clinical characteristics and serum Ceramides **Abbreviations:** NW: normal weight; OB: obesity; BMI: body mass index; SBP: systolic blood pressure; DBP: diastolic blood pressure; WC: waist circumference; ALT: alanine aminotransferase; AST: aspartate aminotransferase; TG: triglyceride; TC: total cholesterol; LDL-c: low-density lipoprotein cholesterol; HDL-c: high-density lipoprotein cholesterol; Lp(a): lipoprotein(a); FPG: fasting plasma glucose; SCr: serum creatinine; BUN: blood urea nitrogen; UA: uric acid The correlation coefficient (from -1 to 1) between each index is presented with the color and size of cell (from red to blue). All the colored cells indicate P<0.005, while the blank ones indicate P≥0.05. Associations between two normally distributed variables were analyzed by Pearson correlation while the others were analyzed by Spearman correlation.

As the serum level of Cer d18:1/24:1 increased, the TC, LDL-c, FPG, TG, SBP, and DBP also increased in parallel. With regard to insulin sensitivity, the levels of Cer d18:1/16:0, Cer d18:0/18:0 and Cer d18:0/24:1 had positive relations with the changes in GIR_30_. Meanwhile, Cer d18:1/16:0 had a positive correlation with HDL-c. The less favorable metabolic phenotypes including high serum TC, TG, LDL-c, AST, ALT, SBP, DBP, WC, and UA, were positively associated with BMI and inversely correlated with the changes in GIR_30_. All the correlations among clinical characteristics are presented in the correlation heatmap (Fig. 2).

### Binary logistic regression in obesity

**Fig. 3:**
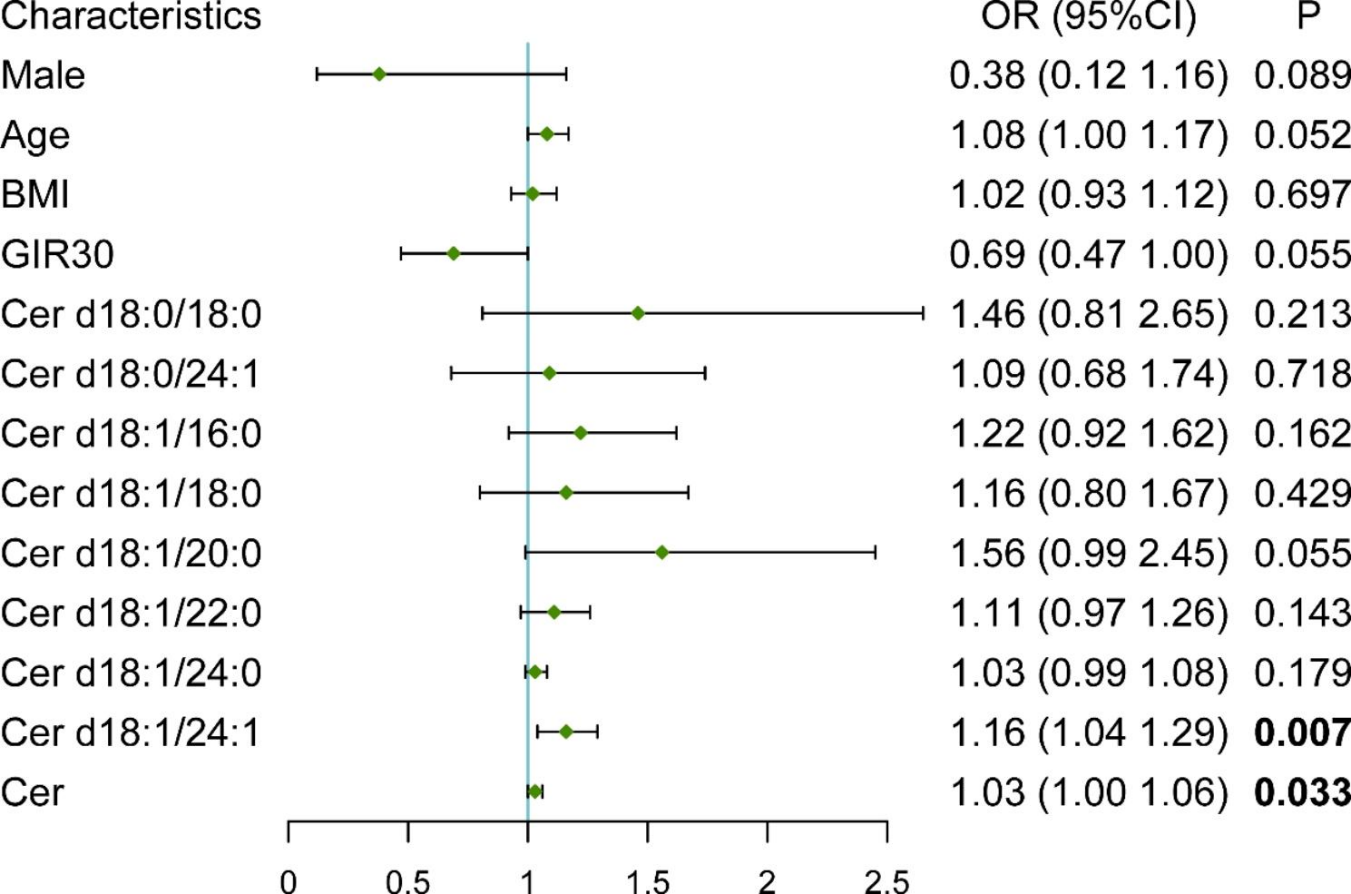
Forest plot of univariate logistic regression of MUO in obesity **Abbreviations:** BMI: Body mass index; Cer: Ceramide The forest plot shows the models of univariate logistic regression in obesity. The concentration values of ceramides were normalized by converting the unit to nmol/cL The lengths of the lines represent the confidence intervals and the green points represent the odds ratio of MUO for each factor in logistic regression

The relationship between serum ceramide levels or demographic parameters and the presence of MUO versus MHO in the obese population (n = 56) was further examined. In the univariate logistic regression models, we found that Cer d18:1/24:1 (OR = 1.16, 95% CI [1.04–1.29], *P* = 0.007) and total ceramides (OR = 1.03, 95% CI [1.00-1.06], *P* = 0.033) contributed to the presence of the MUO phenotype in obesity (Fig. 3). In this study, the obesity subtypes were classified based on metabolic syndrome (MS). Therefore, the parameters in MS were not taken to adjust the models. Age and GIR_30_ were adjusted in a multivariate logistic regression analysis. The results are displayed in Table 3. Cer d18:1/24:1 was independently correlated with MUO in obesity.

**Table 3:** Multivariate logistic regression analysis of MUO in obesity

	Variable	95% CI	OR	P
**Model 1**	Cer d18:1/24:1	[1.03, 1.29]	1.16	**0.012**
	GIR_30_	[0.48, 1.08]	0.72	0.113
**Model 2**	Cer d18:1/24:1	[1.05, 1.32]	1.18	**0.007**
	age	[1.00, 1.17]	1.08	**0.043**
**Model 3**	Cer d18:1/24:1	[1.04, 1.33]	1.17	**0.012**
	GIR_30_	[0.40, 1.01]	0.64	0.057
	age	[1.01, 1.19]	1.10	**0.024**

### Ceramides and obesity related diseases

In addition, the ASCVD risks between different serum levels of ceramides were assessed. Six participants aged 18–20 years old were excluded according to the prediction model. One participant was excluded due to missing data. The 10-year ASCVD risk was generally low as our study population tended to be young. The obese subjects were divided into two groups based on serum levels of Cer d18:1/24:1 which were below or above the median. The lifetime ASCVD risks were significantly higher in the above median group than in the below median group (Table 4). And then 49 obese individuals were divided into four groups based on the CERT1 risk score (the risk of CVDs are classified as low, moderated, increased and high). There’s no difference of China-PAR risk score among four groups (supplementary Fig. 1). To further explore the relationship between Cer d18:1/24:1 and liver fibrosis in NAFLD, we calculated FIB4 and NFS in 47 obese subjects (nine subjects were excluded for missing platelet data). However, we didn’t come to the conclusion since no individual in the study population meet the standard of fibrosis (due to the young age).

**Table 4:** ASCVD risk in obese individuals with different levels of Cer d18:1/24:1

	Group 1 (N = 24) (Mean ± SD)	Group 2 (N = 25) (Mean ± SD)	P
**Sex (Male : Female)**	13:11	8:17	0.117^1^
**age (year)**	31.79 ± 9.55	29.68 ± 7.44	0.391^3^
**BMI (kg/m** ^**2**^ **)**	35.38 ± 4.62	37.75 ± 7.29	0.183^3^
**SBP (mmHg)**	134.58 ± 19.11	143.08 ± 17.28	0.109^3^
**DBP (mmHg)**	83.79 ± 10.48	91.44 ± 13.50	**0.032** ^**3**^
**WC (cm)**	108.71 ± 11.76	108.98 ± 12.88	0.940^3^
**ALT (U/L)**	61.91 ± 50.85	76.09 ± 52.01	0.308^2^
**AST (U/L)**	38.82 ± 24.93	52.52 ± 32.82	0.215^2^
**TG (mg/dL)**	148.96 ± 112.74	188.90 ± 105.28	0.087^2^
**TC (mg/dL)**	187.55 ± 40.93	205.27 ± 33.63	0.104^3^
**LDL-c (mg/dL)**	120.22 ± 34.31	136.39 ± 26.31	0.070^3^
**HDL-c (mg/dL)**	44.33 ± 8.50	41.71 ± 7.79	0.265^3^
**Lp(a) (mg/L)**	185.48 ± 188.97	164.56 ± 184.88	0.952^2^
**GIR**_**30**_	4.70 ± 1.70	4.22 ± 1.43	0.503^2^
**FPG (mg/dL)**	102.01 ± 27.37	112.97 ± 32.83	0.190^2^
**SCr (µmol/L)**	66.26 ± 11.45	60.62 ± 12.61	0.108^3^
**BUN(mmol/L)**	4.59 ± 1.14	4.54 ± 1.08	0.865^3^
**UA(µmol/L)**	407.11 ± 93.64	404.49 ± 93.41	0.922^3^
**10-year ASCVD risk(%)**	1.43 ± 2.60	2.00 ± 2.92	0.477^3^
**Lifetime ASCVD risk (%)**	23.46 ± 9.24	32.6 ± 13.68	**0.009** ^**3**^

Group 1 included obese participants with a Cer d18:1/24:1 level below the median of the study population (898.29-1987.69 nmol/L). Group 2 included obese participants with a Cer d18:1/24:1 level above the median of the study population (1990.50-4491.76nmol/L). The continuous variables between two groups that were normally distributed were compared via Student’s t test

## Discussion

### Ceramides and outcomes of obesity subtypes

Sphingolipids and fatty acids are associated with the pathogenesis of various metabolic diseases. We have previously revealed the novel associations between serum sphingolipid and insulin sensitivity and identified that SM/Cer and SM 18:0/26:0 could be good serum lipid predictors for insulin sensitivity [17]. Most previous studies have explored the relationships between ceramides and obesity. This study explored the roles of ceramide in two obesity subtypes and its impact on obesity related diseases, especially CVD. According to previous studies, there are many classification criteria for MHO and MUO, most of which are based on MS, insulin sensitivity or inflammatory indices [2]. An important stage in the development of MS is insulin resistance. Ceramides bound to LDL stimulate the expressions of inflammatory genes in macrophages, such as IL-6 and TNF, and increase insulin resistance in skeletal myocytes [28]. The review of 2022 suggested that sphingolipid profiling, especially ceramide profiling, was considered as a potential tool for MetS-associated risk stratification [29]. Many studies have proven that activation of β-cell apoptosis is a diabetogenic effect of elevated ceramide levels [30]. Furthermore, specific ceramide subtypes are related to insulin resistance. Early in 2017, dihydroceramide levels, reported as predictors of diabetes, are elevated up to 9 years prior to the onset of diabetes according to a study conducted in two cohorts by Wigger et al. [31]. We have also previously reported the relationships between sphingolipids and insulin sensitivity [17]. In addition to the aforementioned classification criteria for MHO and MUO, some researchers also believed that the different risks of cardiometabolic diseases are the main identified factors for MHO and MUO [5]. As there are many types of ceramide-based scores that can reliably predict the risk or severity of CVDs [24], the relationship between the levels of ceramides and the risks of CVD in Chinese people was examined to prove that ceramide might be a potential biomarker for MUO in this study. Circulating ceramides have been proved to be used as biomarkers for the development and progression of CVD according to the recent studies. They have already been suggested to predict the cardiovascular events more accurately than traditional risk factors, such as LDL-c or HDL-c [32–34].

To fit the clinical characteristics of obesity subtypes, we divided 56 obese individuals into two groups based on the classical definition of MUO (MS standard) [19]. Compared to the NC group, the individuals in either the MHO or MUO group had higher blood glucose, and blood pressure, and unfavorable liver function. Although MHO is regarded as a more favorable subtype than MUO, the health outcomes of MHO remain unclear and controversial. According to previous studies, the risks of metabolic diseases such as T2DM, CVD or NAFLD in individuals with MHO appear to be higher than those in normal-weight individuals [35–38]. Nevertheless, active and effective interventions should be given in a timely manner for individuals with MUO.

### Ceramide subtypes and metabolism

Ceramides are essential elements of biological membranes and signaling molecules involved in a variety of cellular processes [16]. Although the increased levels of ceramides are proved to be associated with obesity, the ceramide subtype levels in MHO and MUO have not been assessed.

In this study, the levels of total ceramides and Cer d18:1/24:1 were higher in MUO than those in MHO. Meanwhile, the correlation between Cer d18:1/24:1 levels and the presence of MUO further indicated that the metabolism of Cer d18:1/24:1 might play an important role in the MUO. To test the relationships between Cer d18:1/24:1 and comorbidities related to obesity, we used the Chinese ASCVD risk prediction equations (China-PAR) to assess cardiovascular outcomes in groups with high or low circulating Cer d18:1/24:1. We found that the circulating Cer d18:1/24:1 level was higher in obese individuals with higher ASCVD risks. Many researches indicated that people in Asian Pacific region, including those in China, have a higher risk developing obesity-related diseases, even when their BMI is lower than that of Caucasians. We conducted our study based on the Chinese standard for obesity [18, 39]. We presumed that certain ceramide might provide similar information as China-PAR to predict cardiovascular events. Similarly, higher circulating Cer d18:1/24:1 levels were found in obese subjects with higher ASCVD risks and had already been proven to be a biomarker of high risk of CVDs according to a large number of studies [8]. Based on previous findings, there are many types of ceramide-based scores that can reliably predict the risk or severity of CVDs, including the ceramide test score (CERT1) [23, 24], the new ceramide test score (CERT2) [34], and the Sphingolipid Inclusive Score (SIC) [40]. The first two calculated scores involve the concentration of Cer d18:1/24:1 and have already been used in Mayo Clinic [24]. In this cross-sectional study, the CERT1 score was also calculated to predict the risks of CVDs. Unfortunately, there’s no significant association between China-PAR risk score and CERT1 score in these obese Chinese population. Although there were some studies on relationships between ceramide levels and risks or severity of CVDs in Chinese population [41, 42], there’s no study validating the efficiency of CERT1 in predicting CVDs in longitudinal perspective Chinese cohort. In addition, the CERT1 was used in general population rather than obese individuals. Further perspective studies are necessary to be conducted to validate the efficiency of CERT1 in Chinese population and to figure out a more suitable prediction model of CVDs based on ceramides. NAFLD is another obesity-related disease especially in MUO. In this study, the hepatic fibrosis scores including FIB4 and NFS were also calculated, but there was no firm conclusion since no individual in this study met the standard of fibrosis due to their young age. However, based on previous studies, a high level of Cer18:1/24:1 was correlated with NAFLD. The review in 2021 summarized the functions of ceramides in the development of liver steatosis and its transition to NASH as well as hepatic fibrosis. Ceramide 18:1/24:1 increased in patients with NASH or insulin resistance [43]. Moreover, ceramide-lowering interventions can resolve CVD and metabolic disorders, including dyslipidemia, hypertension, atherosclerosis, insulin resistance and hepatic steatosis [16]. All of the evidence proved that a high level of Cer 18:1/24:1 was not only related to metabolic disorders of obesity, but also possibly contributed to obesity comorbidities including T2DM, NAFLD, and CVD. It might be an ideal criterion to classify MHO and MUO in the future.

For other subtypes of ceramides, there was no statistical difference between the MHO and MUO groups. Both levels of Cer d18:0/18:0 and Cer d18:1/16:0 were lower in obese individuals than those in the NC group. A previous study showed that C16:0-ceramide is proapoptotic, while the C24-ceramide series is antiapoptotic and proliferative [10]. Moreover, the Cer(18:1/18:0)/Cer(18:1/16:0) ratio can also predict T2DM up to 10 years prior to the development of the disease [44]. A prospective study conducted in patients who had undergone clinical coronary angiography at Mayo Clinic indicated that elevated Cer 18:1/16:0, Cer 18:1/18:0, and Cer 18:1/24:1 levels have predictive value in coronary artery dysfunction [45]. Recent research has demonstrated that plasma Cer 18:0/18:0, which are detected in plasma TG-rich VLDL and elevated with hepatic steatosis, are related to the severity of NAFLD [46]. However, increased Cer d18:1/16:0 and Cer d18:0/18:0 levels were observed in the normal weight group compared to the obese subjects in this study. As the BMI and age of study population for these two studies differ from ours, we assumed that these subtypes of ceramide might play different roles in populations with different ages and body weights. Further prospective studies in various populations are still needed. Recent studies have demonstrated the heritability of plasma concentrations of C18:1/22:0 and C18:1/24:0 which are also associated with the incidence and all-cause mortality of CVD [47]. In this study, Cer 18:1/22:0 and Cer 18:1/24:0 significantly increased compared with the NC group. These data suggested that the subtypes of ceramides might have different impacts on the body metabolic mechanism and the progression of diseases.

### Ceramide-targeting treatment

The study results indicated that more attention should be focused on ceramides because many functions of ceramides are still unknown and should be further explored. Studies in mice demonstrated that the inhibition of ceramide synthesis improved hepatic steatosis and slowed the progression of cardiometabolic diseases [15]. Liraglutide can prevent hepatic inflammation and fibrosis by inhibiting the accumulation of Cer 18:1/16:0 and Cer 18:1/24:0 in the liver of methionine-choline deficient dietary mice [48]. Liraglutide, through inhibiting ceramide levels, may alleviate the adverse effects of cardiac dysfunctions [49]. As most recent studies have targeted the ceramides to treat insulin resistance, fatty liver disease or some comorbidities of obesity [50], further study of ceramide subtypes and mechanisms can be useful to explore specific cures.

### Study strengths and limitations

The study measured the ceramide levels in both MHO and MUO suggesting that the increased level of Cer d18:1/24:1 could be a potential biomarker to differentiate MUO with unfavorable health outcomes from obesity. However, the limitations of this study cannot be ignored. The predominantly Chinese population in this study limits the conclusion generalized to the whole populations. The cross-sectional study design limits the validation of China-PAR and CERT1 risk score in predicting CVD risks in Chinses population. The sample size was small. Some demographic data were collected based on participants recall, which is subject to bias.

## Conclusions

Even though there might be the same risk of developing cardiovascular disease eventually for both MHO and MUO, MUO is worse than MHO due to its rapid progression to poor outcomes. The precise detection of obesity outcomes and early intervention are in need to improve the prognosis. This study showed that the levels of Cer d18:1/24:1 were higher in the MUO group than in the MHO group and were closely associated with higher risks of CVD. The results indicated that the level of Cer d18:1/24:1 might be a potential biomarker to differentiate MUO from MHO, and to better predict the unfavorable health outcomes of MUO. Individuals with obesity with increased levels of Cer d18:1/24:1 might pay attention to losing weight as early as possible. Although our findings are interesting, a large scale, longitudinal, prospective study is still required to further explore and validate the predictive and prognostic value of Cer d18:1/24:1 as a biomarker to differentiate MUO from MHO, and to identify the best cutoff value. Meanwhile, whether ceremide could be considered as a therapeutic target for metabolic disorders of obesity remains unknown.

### Electronic supplementary material

Below is the link to the electronic supplementary material.

Supplementary Material 1

Supplementary Material 2

Supplementary Material 3

Supplementary Material 4

Supplementary Material 5

Supplementary Material 6

## Data Availability

The datasets used and/or analyzed during the current study are available from the corresponding author on reasonable request.

## Abbreviations

T2DM: Type-2 diabetes mellitus
NAFLD: Nonalcoholic fatty liver disease
NASH: Nonalcoholic steatohepatitis
ASCVD: Arteriosclerotic Cardiovascular Disease
CVD: Cardiovascular Disease
BMI: Body mass index
SBP: Systolic blood pressure
DBP: Diastolic blood pressure
WC: Waist circumference
FPG: Fasting plasma glucose
ALT: Alanine aminotransferase
AST: Aspartate aminotransferase
TC: Total cholesterol
TG: Triglyceride
HDL⁃c: High-density lipoprotein cholesterol
LDL⁃c: Low-density lipoprotein cholesterol
VLDL-c: Very-low-density lipoprotein
Lp(a): Lipoprotein(a)
SCr: Serum Creatinine
BUN: Blood Urea nitrogen
UA: Uric acid
MS: Metabolic syndrome
SLs: Sphingolipids
Cer: Ceramide
MHO: Metabolically healthy obesity
MUO: Metabolically unhealthy obesity
NC: Normal control
GIR: Rate of glucose infusion
GDR: Rate of glucose disposal
NFS: Nonalcoholic fatty liver disease score
FIB4: Fibrosis-4
GLUT4: Glucose Transporter 4
OR: Odds ratio
CRP: C-reactive protein
TNF-α: Tumor necrosis factor Alpha
GLP-1: Glucagon-like peptide-1
