# Supplementary material for: Ceramide d18:1/24:1 as a potential biomarker to differentiate obesity subtypes with unfavorable health outcomes
Source: Lipids Health Dis. 2023 Oct 4;22:166. doi: 10.1186/s12944-023-01921-0 (PMC10548646; doi:10.1186/s12944-023-01921-0)
Supplement: Supplementary file 1 — Supplementary Material 1 [file 12944_2023_1921_MOESM1_ESM.pdf]

SPRINGER NATURE

Author Services

Editing Certificate

This document certifies that the manuscript

Ceramide d18:1/24:1 as a potential biomarker to differentiate the obesity subtypes with unfavorable health outcomes

prepared by the authors

Baowen Yu, Moran Hu, Wanzi Jiang, Yizhe Ma, Jingya Ye, Qinyi Wu, Wen Guo, Yan Sun, Min Zhou, Yiwen Xu, Zhoulu Wu, Yiwen Wang, Sin Man Lam, Guanghou Shui, Jingyu Gu, John Zhong Li, Zhenzhen Fu, Yingyun Gong, Hongwen Zhou

was edited for proper English language, grammar, punctuation, spelling, and overall style by one or more of the highly qualified native English speaking editors at SNAS.

This certificate was issued on **August 3, 2023** and may be verified on the SNAS website using the verification code **4563-AFAD-B14E-4F9D-A878** .

Neither the research content nor the authors' intentions were altered in any way during the editing process. Documents receiving this certification should be English-ready for publication; however, the author has the ability to accept or reject our suggestions and changes. To verify the final SNAS edited version, please visit our verification page at secure.authorservices.springernature.com/certificate/verify.
If you have any questions or concerns about this edited document, please contact SNAS at support@as.springernature.com.

SNAS provides a range of editing, translation, and manuscript services for researchers and publishers around the world.
For more information about our company, services, and partner discounts, please visit authorservices.springernature.com.
