# Supplementary material for: Ceramide d18:1/24:1 as a potential biomarker to differentiate obesity subtypes with unfavorable health outcomes
Source: Lipids Health Dis. 2023 Oct 4;22:166. doi: 10.1186/s12944-023-01921-0 (PMC10548646; doi:10.1186/s12944-023-01921-0)
Supplement: Supplementary file 3 — Supplementary Material 3 [file 12944_2023_1921_MOESM3_ESM.pdf]

Ceramide d18:1/24:1 as a potential biomarker to...

By: Baowen Yu

As of: Aug 7, 2023 7:40:01 AM
4,125 words - 87 matches - 64 sources

Similarity Index

23%

Mode: Similarity Report

paper text:

Ceramide d18:1/24:1 as a potential biomarker to differentiate obesity subtypes with unfavorable health outcomes Abstract Background: The criteria for

metabolically healthy obesity (MHO) and metabolically unhealthy obesity (MUO 18

) remain controversial. This research aimed to identify a potential biomarker to differentiate the subtypes of obesity. Methods: The study conducted a lipidomic evaluation of ceramide in the serum of 77 Chinese adults who had undergone hyperinsulinemic-euglycemic clamps. These adults

were divided into three groups according to the clinical data: **normal** weight control **group** 30

(NC, N=21), MHO (N=20) and MUO (N=36). Results: The serum Cer d18:1/24:1 level

in the MHO **group was lower than that in the** MUO **group** . As **the** 11

Cer d18:1/24 :1 **level increased** 8

, insulin sensitivity decreased, and the unfavorable parameters increasedin parallel.

Multivariate logistic regression analysis revealed that 4

serum

Cer d18:1/24:1 levels were independently correlated **with** 52

MUO in obesity. Individuals with higher

levels of Cer d18:1/24:1 also had **an** elevated **risk of cardiovascular** 27

disease. Most ceramide subtype levels increased in obesity compared to normal-weight individuals, but the

levels of serum **Cer d18** :0/ **18:0 and Cer d18:1** /16: **0** decreased **in** obesity. 1 Conclusions: **The** 9

relationships between ceramide subtypes and metabolic profiles might be heterogeneous in populations with different body weights. Cer d18:1/24:1 could be a biomarker that can be used to differentiate MUO from MHO, and to better predict who will develop unfavorable health outcomes among obese individuals. Key words: metabolically unhealthy obesity, ceramide, Cer d18:1/24:1, cardiovascular disease Background Obesity now represents a worldwide public health problem, impacting the health of a significant number of peopleworldwide.

107.7 million children and 603.7 million adults were identified as **obese in 2015** according to **the** 39

Global Burden of Disease Obesity Collaborators 64

. Approximatedly 4.0 million deaths, mostly due to cardiovascular disease (CVD), are related tohigh

body mass index (BMI) [1]. **These** findings indicated **the** 4

importance of effective treatments for decreasing obesity prevalence and disease burdens. Obesity is associated with many kinds of metabolic abnormalities and

diseases, including type 2 diabetes mellitus (T2DM), nonalcoholic fatty liver disease (NAFLD), and CVD 38

[2]. Obese individuals who exhibit fewer metabolic dysfunctions are considered metabolically healthy obesity (MHO), and they may represent a unique group or a group that is in the process of transitioning to metabolically unhealthy obesity (MUO) [3]. In contrast, MUO was reported as a less favorable type of obesity with impaired lipid and glucose

metabolism and a high risk of cardiovascular and inflammatory abnormalities and other metabolic disorders [4]. There are many definitions for MUO, most of which are based on phenotypes with unfavorable laboratory findings, such as inflammatory markers, metabolic parameters, insulin sensitivity, fibrinolytic activity, and liver function [2]. Some researchers also believed that the different risks of cardiometabolic diseases are the main identified factors for MHO and MUO[5]. However, the various definitions of MHO and MUO are still controversial [3]. The current classifications lack standardization and may not be sufficient or accurate to identify the specific obese subgroup. There is thus a need to have a better biological marker that can make the classification of obesity more accurate, with better prediction of health outcomes so that interventions can be initiated earlier to improve the disease outcomes. Sphingolipids (SLs), the minor components of membranes, have

crucial biological functions such as altering **the** physiochemical characteristics **of** lipid bilayers **and** 56

influencing the activation of intracellular proteins and receptors. Although SLs comprise only 2-15% of the total cellular lipidome, they play important roles in the development of metabolic diseases and CVD [6]. Ceramide, one of the best characterized SLs, represents a heterogeneous group of lipids that are identified by the specific fatty acyl moiety bonded to sphingosine with an amide bond. The different fatty acyl moieties encompass short to long fatty acids (C2-C34) [7]. Ceramides, acting as secondary messengers for cellular signaling, are related to both lipid and glucose metabolism [8]. As reported before, ceramides were proven to play crucial roles in cell proliferation, autophagy, apoptosis, senescence, migration, regulating mitochondrial dynamics, lipid utilization, glucose sensitivity and inflammation [9-11]and were associated with depression [12], cancer [13], and neurodegenerative disorders [14]. However, in recent years, ceramides are getting increased attention for their important roles in metabolic dysfunctions such as obesity, hepatic steatosis, diabetes, and CVD [15]. Although increased levels of ceramides were shown to be associated with obesity [16], no study has assessed ceramides levels with regard to MHO or MUO. This study measured the ceramide levels in both MHO and MUO to evaluate the efficiency of ceramides in differentiating the subtypes of obesity with unfavorable health outcomes. Methods Data source. Data were derived from a cohort of participants who had undergone both hyperinsulinemic-euglycemic clamp and lipidomic analysis

in the First Affiliated Hospital of Nanjing Medical University, Nanjing, China [17]. **The** 46

participants who underwent the hyperinsulinemic-euglycemic clamp had not taken any medication or supplement, had no history of smoking or high alcohol intake (four

or more standard drinks per **week for men and** and two **or more standard drinks** per **week for women** 35

), severe disease, acute inflammation or pregnancy. Individuals with blood samples of poor quality and missing physical examination data were excluded when the lipidomic analysis was conducted. We assessed

90 lipid species in 7 classes of lipids, including 2

free fatty acids, sphingomyelins,

ceramides, glu-ceramides, lac-ceramides, ganglioside and globotriaosylceramides 1

. Study population. The eligibility criteria for the study included normal weight or obese individuals aged 18-55 years old. Obesity and normal weight were defined as a BMI ≥ 28 kg/m2 and 18.5-23.9 kg/m2 respectively based on Chinese standards[18]. We excluded subjects whose ceramide subtype concentrations were below the limit of detection. A total of 77 individuals were finally enrolled. Demographic characteristics and physical examination data included sex, age, geographic region,

body mass index (BMI), systolic/diastolic blood pressure (SBP/DBP) and waist circumference (WC 20
). These individuals **were**

divided into three groups based on their BMI and metabolic parameters: normal weight control (NC) group, MHO and MUO. None of the individuals in the NC group had evidence of hypertension, hyperlipidemia, diabetes, or any other diseases. Obese

individuals who met at least three of the following criteria were defined as 23

MUO based on metabolic syndrome: 1. 1.

WC ≥90 cm in men or ≥80 cm in women (recommended by WHO **for** WC Threshold **for** 34
abdominal **obesity in**

Asian); 2.

Fasting serum triglycerides ≥150 mg/dL (1.7 mmol/L) or receiving lipid- **lowering** 47

treatment;

3. HDL -C ≤ **40 mg/dL (1.0 mmol/L**) in **men** or ≤ **50 mg/dL (1.3 mmol/L**) in **women;** 6
4 . SBP ≥ **130 mmHg and/or** DBP ≥ **85 mmHg or** receiving **antihypertensive** treatment;
and **5.Fasting** glucose ≥ **100 mg/dL**

or diagnosed with diabetes [19]. The other individuals were classified as MHO.

Hyperinsulinemic-euglycemic clamp. The hyperinsulinemic-euglycemic clamp 7

method established by DeFronzo et al. was used

to assess whole-body insulin sensitivity 63

. [20]. The specific clamp test details were described previously [17]. The hepatic glucose production could be suppressed

by a high dose of insulin use (> **80 mU/m2*min** 26

). When the circulation blood glucose is stable,

the rate of glucose infusion (GIR) is equals **to that of whole-body glucose disposal (GDR** 1

) representing body insulin sensitivity. After a preparation of insulin injection, the body can

achieve a steady-state insulin concentration. The 7

glucose infusion ensures the plasma glucose concentration at

approximately 5 mmol/L. The mean glucose infusion rate for **the last 30** minutes **was** 41

calculated as GIR30 representing body insulin sensitivity. Laboratory measurements. The assessments included plasma glucose (using

a blood glucose biochemical analyzer -Germany, Biosen 7

). Standard enzymatic assays were

used to measure total cholesterol (TC), triglyceride (TG), low-density lipoprotein cholesterol (LDL-c), high-density lipoprotein cholesterol (HDL-c 28

) and other biochemical phenotypes

in the laboratory of the First Affiliated Hospital of Nanjing Medical University, Nanjing, China 2

. Measurements of ceramide levels. The lipidomic lipid methods were reported previously [17].

Lipids were acquired **from serum (20 μL**) and dried in **a** 7

SpeedVac of OH mode

according to a modified version of **Bligh and Dyer's extraction** method (**double rounds of extraction**). Lipid **extracts were** redissolved **in** 32

a 1:1 (v/v) solution of chloroform: methanol spiked with relevant internal standards before analyzing. The

lipidomic analyses were conducted **on an Exion UPLC system coupled with a QTRAP 6500 PLUS system (Sciex** 2

).

Sphingolipids were separated on a Phenomenex Luna Silica 3 μm column (i.d. 150x2.0 mm). The chromatographic conditions are as follows: **mobile phase A (chloroform:methanol:ammonium hydroxide, 89.5:10:0.5) and mobile phase B (chloroform:methanol: ammonium hydroxide: water,** 1

55:39:0.5:5.5) at a flow rate of 270 μL/min and column oven temperature of 25 °C. Individual sphingolipid species were quantified by reference to spiked internal standards including Cer d18:1/17:0, GluCer d18:1/8:0, LacCer d18:1/8:0, and SM d18:1/12:0, obtained from Avanti Polar Lipids; d3-GM3 d18:1/18:0 and Gb3 d18:1/17:0 purchased from Matreya LLC; d8-FFA 20:4 from Cayman Chemicals; and d31-FFA16:0 from Sigma Aldrich

. Risk assessment models.

The risk of arteriosclerotic **cardiovascular** disease (ASCVD) **was assessed** by **the** prediction model **of** 13

China-PAR. The total study sample size of the China-PAR project was more than 127 thousand and the longest follow-up time was more than 23 years[21,22]. This effective tool has already been proven to have good performance in predicting ASCVD risk in the Chinese population[21,22]. The risks of individuals were assessed using the following website: http://www.cvdrisk.com.cn (10-year ASCVD risk: high risk≥10. 0%, medium risk: 5.0%~9.9%, low risk<5.0%). Lifetime ASCVD risk was defined as follows: the risk of developing ASCVD from now to 85 years old, with the cutoff for low and high risk set to 32.8%.) [21,22].

Fibrosis-4 (FIB-4) and nonalcoholic **fatty liver disease score (NFS) were** 49

used to assess hepatic fibrosis.

FIB-4= [age (years) × AST(U/L)]/[PLT (×109/L) ×√ALT (U/L)]. The 37

cutoff values for stage 0-2 hepatic fibrosis and significant fibrosis are <1.3 and >2.67 respectively[23,24].

NFS = − 1.675 + 0.037 × age (y) + **0.094 × BMI (kg/m2) + 1.13 × impaired fasting glucose or diabetes (yes=1, no=0) + 0.99 × AST/ALT ratio − 0.013 × platelet count (×109/L) −0.66 × albumin (g/dL). The** 3

presence of advanced fibrosis was detected **with** good **accuracy** 29

using the high cut-off point 29

of NFS (0.676), while it was excluded using

the low cut-off point (-1.455 29

) of NFS. [25]. Statistics.

Continuous variables are represented **as** the **mean** ± standard deviation (**SD) or median (quartile 1, quartile 3), and categorical variables** are represented **as** 31

the frequency. Individuals with values of variables above or below the mean ± 5 SD were regarded as outliers, and these individuals were excluded. The

Mann–Whitney U test was used **for data** that were non- **normally distributed** , while **Student's t test** was used **for data** that were **normally distributed** when **the** differences **between** two **groups were analyzed. The** Kruskal–Wallis **test** 24

was used for data that were non- **normally distributed** 60

, while one-way ANOVA was used for data normally distributed when multiple group differences

were analyzed. Categorical variables were compared by **the chi-square test** 16

. The relationships between ceramides and metabolic characteristics were analyzed

by Pearson correlation (normally distributed data) or Spearman correlation (non-normally distributed data 42

). Models of binary

logistic regression were fitted to estimate the associations between ceramides and the presence 4

of MUO. The software IBM SPSS 22.0 and statistical software R 4.1.0 were

used to conduct the statistical analysis. Statistical significance was defined as P<0.05 15

. Study approval.

This study approval was granted by the Institutional Review Board of the First Affiliated Hospital of Nanjing Medical University, and all participants provided written informed consent (2014-SR-003 2

) prior to study entry. Results Clinical characteristics of the subjects. Among the 77 individuals with BMI 18.73-23.8kg/m2 (normal weight) and 28.01-61.91kg/m2 (obese), 21 (27%) were classified as the NC group, 20 (26%) were classified as MHO and another 36 (47%) were classified as MUO. Basic clinical characteristics based on metabolic phenotypes are displayed in Table 1(the proposed normal ranges of values given by the laboratory for blood parameters are displayed in Supplementary

Table 1). There was no statistical difference in BMI between the MHO and MUO groups. The 25

distributions of sexes were not

significantly different among the three groups . GIR30 (which 4

represents whole body insulin sensitivity. see methods.) tended to decrease in MHO/MUO

groups compared to the NC group. However , GIR30 showed no significant 4

difference between MHO and MUO. LDL-c, the biomarker to assess CVD risk, increased significantly in both MHO and MUO. In general, all the metabolic parameters

in the obesity group were worse than those in the NC group . Ceramides among the three groups. The 43

concentrations of ceramides were logarithmically transformed into a normal distribution.

The differences in ceramide levels **among the** three **groups** are shown **in** Table 2. **The** 54

heatmap of ceramides (Fig. 1A) visualizes the concentrations. The level

of Cer d18:1/24:1 was lower 58

in both the NC and MHO groups than in the MUO group. Total serum ceramide showed asimilar trend

to Cer d18:1/24 :1. Most notably, **there** were **significant** 8

increases in serum

Cer d18 :0/ **18:0 and Cer d18:1** /16: **0** levels **in the** NC group compared **with the** 9

MHO group. We also compared

the levels of Cer d18:0 /18: **0 and Cer d18:1** /16: **0** 48

between normal weight and obese individuals. Our results showed

that the levels of Cer d18 :0/18: **0 and Cer d18:1** /16: **0** were **significantly** lower **in** 36

people with obesity. The obese

individuals were then **divided into** 5 groups **on the basis of** 59

the numbers of metabolic risk abnormalities (Method:five criteria of metabolic syndrome) (Fig. 1B). The percentage of individuals with two to four metabolic abnormalities was 76.8%. There is no significance different distribution of genders among 5 groups 10 (P=0.435).The level of Cer d18:1/24:1 showed an increasing trend with the accumulation of abnormality numbers (Fig. 1C). Correlations between Ceramides and multiple clinical characteristics. As

the serum level **of Cer d18:1/24:1** increased, **the** 53

TC, LDL-c,FPG, TG, SBP, and DBPalso increased in parallel. With regard to insulin sensitivity,

the levels of Cer d18:1/16:0, Cer d18 :0/ **18:0 and Cer d18** :0/ **24:1** 14

had positive relations with the changes in GIR30. Meanwhile,

Cer d18:1 /16: **0 had** a positive correlation **with** HDL- **c** 62

). The less favorable metabolic phenotypes including high serum TC, TG, LDL-c, AST, ALT, SBP, DBP, WC, and UA,

were positively associated with BMI **and inversely** correlated **with** the changes **in** GIR30. All **the** 12

correlations among clinical characteristics are presented in the correlation heatmap (Fig. 2). Binary logistic regression in obesity. The relationship between serum ceramide levels or demographic parameters and the presence of MUO versus MHO in the obese population (n=56) was further examined.

In the univariate **logistic regression models, we found that** 19

Cer d18:1/24:1 (

OR=1.16, 95% CI [1.04-1 .29], **P=0** .007) **and** total ceramides (**OR=1** .03, **95% CI [1** 10

.00- **1** .06], **P=0**

.033) contributed to the presence of the MUO phenotype in obesity (Fig. 3). In this study, the obesity subtypes were classified based on metabolic syndrome (MS). Therefore, the parameters in MS were not taken to adjust the models. Age and GIR30

were adjusted in a multivariate **logistic regression analysis. The results** are displayed **in** 5

Table 3. Cer d18:1/24:1 was independently correlated with MUO in obesity. Ceramides and obesity related diseases. In addition, the ASCVD risks between different serum levels of ceramides were assessed. Six participants aged 18-20 years old were excluded according to the prediction model. One participant was excluded due to missing data. The 10-year ASCVD risk was generally low as our study population tended to be young. The obese subjects

were divided into two groups based on serum **levels** 30

of Cer d18:1/24:1 which were below or above the median. The lifetime ASCVD risks

were significantly higher **in the** above median **group than** in **the** below median **group (Table 4**). To further explore **the** 50

relationship between Cer d18:1/24:1 and liver fibrosis in NAFLD, we calculated FIB4 and NFS in 47 obese subjects (nine subjects were excluded for missing platelet data). However, we didn’t come to the conclusion since no individual in the study population meet the standard of fibrosis (due to the young age). Discussion Ceramides and outcomes of obesity subtypes Most previous studies have explored the relationships between ceramides and obesity. This study explored the roles of ceramide in two obesity subtypes and its impact on obesity related diseases, especially CVD. According to previous studies, there are many classification criteria for MHO and MUO, most of which are based on MS, insulin sensitivity or inflammatory indices [2]. A important stage in the development of MS is insulin resistance. Ceramides bound to LDL stimulate the expressions of inflammatory genes in macrophages, such as IL-6 and TNF, and increase insulin resistance in skeletal myocytes [26]. The review of 2022 suggested that sphingolipid profiling, especially ceramide profiling, was considered as a potential tool for MetS-associated risk stratification[27]. Many studies have proven that activation of β-cell apoptosis is a diabetogenic effect of elevatedceramide levels [28]. Furthermore, specific ceramide subtypes are related to insulin resistance . Early in 2017, dihydroceramide levels, reported as predictors of diabetes, are elevated up to 9 years prior to the onset of diabetes according to a research conducted in two cohort studies by Wigger et al [29]. We have also previously reported the relationships between sphingolipids and insulin sensitivity [17]. In addition to the aforementioned classification criteria for MHO and MUO, some researchers also believed that the different risks of cardiometabolic diseases are the main identified factors for MHO and MUO [5]. As

there are many types of ceramide-based scores that can reliably predict the risk or severity of CVDs [30], the relationship between the levels of ceramides and the risks of CVD in Chinese people was examined to prove that ceramide might be a potential biomarker for MUO in this study. Circulating ceramides have been proved to be used as biomarkers for the development and progression of CVD according to the recent studies. They have already been suggested to predict the cardiovascular events more accurately

than traditional risk factors, such as LDL-c 57

or HDL-c [31-33]. To fit the clinical characteristics of obesity subtypes, we divided 56 obese individuals into two groups based on the classical definition of MUO (MS standard) [19].

Compared to the NC **group** , the individuals **in** either **the MHO** or MUO **group had higher** 21

blood glucose, and blood pressure, and unfavorable liver function. Although MHO is regarded as a more favorable subtype than MUO, the health outcomes of MHO remain unclear and controversial. According to previous studies, the risks of metabolic diseases such as T2DM, CVD or NAFLD in individuals with MHO appear to be higher than those in normal-weight individuals [34-37]. Nevertheless, active and effective interventions should be given in a timely manner for individuals with MUO. Ceramide subtypes and metabolism Ceramides are essential elements of biological membranes and signaling molecules involved in a variety of cellular processes [16]. Although the increased levels of ceramides are proved to be associated with obesity, the ceramide subtype levels in MHO and MUO have not been assessed. In this study, the levels of total ceramides and

Cer d18:1/24:1 were higher in MUO **than those in** MHO. Meanwhile, **the** 17

correlation

between Cer d18:1/24:1 levels and the presence **of** 27

MUO further indicated that the metabolism of

Cer d18:1/24 :1 might **play** an important **role in the** MUO. **To** 55

test the relationships between Cer d18:1/24:1 and comorbidities related to obesity, we used the Chinese ASCVD risk prediction 14 equations (China-PAR) to assess cardiovascular outcomes in groups with high or low circulating

Cer d18:1/24:1 . We found that **the** circulating **Cer d18:1/24** 51

:1 level was higher in obese individuals with higher ASCVD risks. Many researches indicated that people in Asian Pacific region, including those in China, have a higher risk developing obesity-related diseases, even when their BMI is lower than that of Caucasians. We conducted our study based on the Chinese standard for obesity [18,38]. We presumed that certain ceramide might provide similar information as China-PAR to predict cardiovascular events. Similarly, higher circulating

Cer d18:1/24:1 levels were found **in** obese **subjects with** 5

higher ASCVD risks and had already been proven to be a biomarker of high risk of CVDs according to a large number of studies [8]. Based on previous findings, there are many types of ceramide-based scores that can reliably predict the risk or severity of CVDs, including the ceramide test score (CERT1) [30,39], the new ceramide test score (CERT2) [33], and the Sphingolipid Inclusive Score (SIC) [40]. The first two calculated scores involve the

concentration of Cer d18:1/24:1 and 5

have already been used in Mayo Clinic[30]. NAFLD is another obesity-related disease especially in MUO. In this study, the hepatic fibrosis scores including FIB4 and NFS were also calculated, but there was no firm conclusion since no individual in this study met the standard of fibrosis due to their young age. However, based on previous studies, a high level of Cer18:1/24:1 was correlated with NAFLD. The review in 2021 summarized the functions of ceramides in the development of liver steatosis and its transition to NASH as well as hepatic fibrosis. Ceramide 18:1/24:1 increased in 15 patients with NASH or insulin resistance [41]. Moreover, ceramide-lowering interventions can resolve CVD and metabolic disorders, including dyslipidemia, hypertension, atherosclerosis, insulin resistance and hepatic steatosis [16]. All of the evidence proved that a high level of Cer 18:1/24:1 was not only related to metabolic disorders of obesity, but that is also possibly contributed to obesity comorbidities including T2DM, NAFLD, and CVD. It might be an ideal criterion to classify MHO and MUO in the future. For other subtypes of ceramides,

there was no statistical difference between the MHO and MUO **groups** 61

. Both

levels of Cer d18:0 /18: **0 and** Cer d18:1/ **16:0 were** lower **in** obese individuals **than** **in** 40

theNC group.A previous study showed that C16:0-ceramide is proapoptotic, while the C24-ceramide series is antiapoptotic and proliferative [10]. Moreover, the Cer(18:1/18:0)/Cer(18:1/16:0) ratio can also predict T2DM up to 10 years prior to the development of the disease[42]. A prospective study conducted in patients who had undergone clinical coronary angiography at Mayo Clinic indicated that elevated

Cer 18:1/16:0, Cer 18:1/18:0 , and **Cer 18:1/24:1** 33

levels have predictive value in coronary artery dysfunction [43]. Recent research has demonstrated that plasma Cer 18:0/18:0, which are detected in plasma TG-rich VLDL and elevated with hepatic steatosis, are related to the severity of NAFLD [44]. However, increased

Cer d18:1/16:0 and Cer d18 :0/ **18:0** levels **were** observed **in the** 22

normal weight group compared to the obese subjects in this study. As the BMI and age of study population for these two studies differ from ours, we assumed that these subtypes of ceramide 16 might play different roles in populations with different ages and body weights. Further prospective studies in various populations are still needed. Recent studies have demonstrated the heritability of plasma concentrations of C18:1/22:0 and C18:1/24:0 which are also associated with the incidence and all-cause mortality of CVD [47]. In this study,

Cer 18:1/22:0 and **Cer 18:1/24:0 significantly increased** 45

compared with the NC group. These data suggested that the subtypes of ceramides might have different impacts on the body metabolic mechanism and the progression of diseases. Ceramide-targeting treatment The study results indicated that more attention should be focused on ceramides because many functions of ceramides are still unknown and should be further explored. Studies in mice demonstrated that the inhibition of ceramide synthesis improved hepatic steatosis and slowed the progression of cardiometabolic diseases [15]. Liraglutide can prevent hepatic inflammation and fibrosis by inhibiting the accumulation of Cer 18:1/16:0 and Cer 18:1/24:0 in the liver of methionine-choline deficient dietary mice[49]. Liraglutide, through inhibiting ceramide levels, may alleviate the adverse effects of cardiac dysfunctions[45]. As most recent studies have targeted the ceramides to treat insulin resistance, fatty liver disease or some comorbidities of obesity [50], further study of ceramide subtypes and mechanisms can be useful to explore specific cures.

Study strengths and limitations The study measured **the** 8

ceramide levels in both MHO and MUO suggesting that the increased

level of Cer d18:1/24:1 8

could be a potential biomarker to differentiate MUO with unfavorable health outcomes from obesity. However, the limitations of this study cannot be ignored. The predominantly Chinese population in this study limits the conclusion generalized to the whole populations. The sample size was small. Some demographic data were collected based on participants recall, which is subject to bias. Conclusions Even though there might be the same risk of developing cardiovascular disease eventually for both MHO and MUO, MUO is worse than MHO due to its rapid progression to poor outcomes. The precise detection of obesity outcomes and early intervention are in need to improve the prognosis. This study showed that the levels of

Cer d18:1/24 :1 **were higher in the** MUO **group than in the** 44

MHO group and were closely associated with higher risks of CVD.

The results indicated **that** the **level of Cer d18:1/24:1** 5

might be a potential biomarker to differentiate MUO from MHO, and to better predict the unfavorable health outcomes of MUO. Individuals with obesity with increased levels of Cer d18:1/24:1 might pay attention to losing weight as early as possible. Although our findings are interesting, a large scale, longitudinal, prospective study is still required to further explore and validate the predictive and prognostic value of Cer d18:1/24:1 as a biomarker to differentiate MUO from MHO, and to identify the best cutoff value. Meanwhile, whether ceremide could be considered a therapeutic target for metabolic disorders of obesity remains unknown. 18 1 2 3 4 5 6 7 8 9 10 11 12 13 14 15 16 17 18 19 20 21 22 23 24 25 26 27 28 29 30 31 32 33 34 35 36 37 38 39 40 41 42 43 44 45 46 47 48 49 50 51 52 53 54 55 56 57 58 59 60 61 62 63 64 65 66 67 68 69 70 71 72 73 74 75 76 77 78 79 80 81 82 83 84 85 86 87 88 89 90 91 92 93 94 95 96 97 98 99 100 101 102 103 104 105 106 107 108 109 110 111 112 113 114 115 116 117 118 119 120 121 122 123 124 125 126 127 128 129 130 131 132 133 134 135 136 137 138 139 140 141 142 143 144 145 146 147 148 149 150 151 152 153 154 155 156 157 158 159 160 161 162 163 164 165 166 167 168 169 170 171 172 173 174 175 176 177 178 179 180 181 182 183 184 185 186 187 188 189 190 191 192 193 194 195 196 197 198 199 200 201 202 203 204 205 206 207 208 209 210 211 212 213 214 215 216 217 218 219 220 221 222 223 224 225 226 227 228 229 230 231 232 233 234 235 236 237 238 239 240 241 242 243 244 245 246 247 248 249 250 251 252 253 254 255 256 257 258 259 260 261 262 263 264 265 266 267 268 269 270 271 272 273 274 275 276 277 278 279 280 281 282 283 284 285 286 287 288 289 290

291 292 293 294 295 296 297 298 299 300 301 302 303 304 305 306 307 308 309 310 311 312 313 314 315 316 317 318 319 320 321 322 323 324 325 326 327 328 329 330 331 332 333 334 335 336 337 338 339 340 341 342 343 344 345 346 347 348 349 350 351 352 353 354 355 356 357 358 359 360 361 362 363 364 365 366 367 368 369 370 371 372 373 374 375 376 377 378 2 3 4 5 6 7 8 9 11 12 13 17

sources:

1 151 words / 3% - Crossref
Jingya Ye, Xuan Ye, Wanzi Jiang, Chenyan Lu et al. "Targeted lipidomics reveals associations between serum sphingolipids and insulin sensitivity measured by the hyperinsulinemic-euglycemic clamp", Diabetes Research and Clinical Practice, 2021

2 66 words / 1% - Internet from 11-Dec-2022 12:00AM
www.biorxiv.org

3 39 words / 1% - Crossref
Goh Eun Chung, Jeong-Hoon Lee, Heesun Lee, Min Kyung Kim et al. "Nonalcoholic fatty liver disease and advanced fibrosis are associated with left ventricular diastolic dysfunction", Atherosclerosis, 2018

4 36 words / 1% - Crossref
"Posters (Abstracts 301-2389)", Hepatology, 2018

5 35 words / 1% - Internet from 11-Mar-2023 12:00AM
lipidworld.biomedcentral.com

6 32 words / 1% - from 04-Jun-2023 12:00AM
link.springer.com

7 28 words / 1% - Crossref Posted Content
Jingya Ye, Xuan Ye, Wanzi Jiang, Chenyan Lu et al. "Target lipidomics reveals associations between serum sphingolipids and insulin sensitivity by the glucose clamp", Cold Spring Harbor Laboratory, 2020

8 27 words / 1% - Crossref
Chenchen Tu, Lan Xie, Zhenjie Wang, Lili Zhang, Hongmei Wu, Wei Ni, Caixia Li, Lin Li, Yong Zeng. "Association between ceramides and coronary artery stenosis in patients with coronary artery disease", Lipids in Health and Disease, 2020

9 26 words / 1% - Internet from 01-Aug-2021 12:00AM
coek.info

10 19 words / < 1% match - from 24-May-2023 12:00AM
www.science.gov

11 11 words / < 1% match - from 17-Jun-2023 12:00AM
www.science.gov

12 9 words / < 1% match - from 15-Mar-2023 12:00AM
www.science.gov

13 8 words / < 1% match - from 19-Mar-2023 12:00AM
www.science.gov

14 17 words / < 1% match - from 01-Jun-2023 12:00AM
www.mdpi.com

15 14 words / < 1% match - Internet from 19-Oct-2022 12:00AM
www.mdpi.com

16 9 words / < 1% match - Internet from 03-Mar-2023 12:00AM
www.mdpi.com

17 12 words / < 1% match - Internet from 05-Feb-2023 12:00AM
www.researchgate.net

18 9 words / < 1% match - Internet from 26-Jan-2023 12:00AM
www.researchgate.net

19 8 words / < 1% match - Internet from 24-Feb-2023 12:00AM
www.researchgate.net

20 15 words / < 1% match - from 30-May-2023 12:00AM
www.frontiersin.org

21 10 words / < 1% match - from 03-Jun-2023 12:00AM
www.frontiersin.org

22 13 words / < 1% match - Internet
Arterioscler Thromb Vasc Biol. 2019 Nov 26; 39(11):2338-2352

23 11 words / < 1% match - Internet
Hui Liu, Weiquan Lin, Kexin Tu, Qin Zhou, Chang Wang, Minying Sun, Yaohui Li, Xiangyi Liu, Guozhen Lin, Sidong Li, Wei Bao. "Prevalence, awareness, treatment, and risk factor control of high atherosclerotic cardiovascular disease risk in Guangzhou, China", Frontiers in Cardiovascular Medicine

24 23 words / < 1% match - Internet from 09-Feb-2023 12:00AM
acamedicine.org

25 11 words / < 1% match - from 28-Mar-2023 12:00AM
www.researchsquare.com

26 10 words / < 1% match - Internet from 02-Nov-2020 12:00AM
www.researchsquare.com

27 20 words / < 1% match - Crossref
Alessandro Mantovani, Clementina Dugo. "Ceramides and risk of major adverse cardiovascular events: A meta-analysis of longitudinal studies", Journal of Clinical Lipidology, 2020

28 20 words / < 1% match - Internet from 27-Sep-2022 12:00AM
www.spandidos-publications.com

29 19 words / < 1% match - Crossref
Yoshio Sumida, Masato Yoneda, Hideyuki Hyogo, Yoshito Itoh et al. "Validation of the FIB4 index in a Japanese nonalcoholic fatty liver disease population", BMC Gastroenterology, 2012

30 17 words / < 1% match - Internet from 04-Feb-2023 12:00AM
rssdi.in

31 16 words / < 1% match - Crossref
Adolfo Díez-Pérez. "Risk factors for prediction of inadequate response to antiresorptives", Journal of Bone and Mineral Research, 04/2012

32 16 words / < 1% match - Crossref
Xiaojing Wang, Sin Man Lam, Mingjun Cao, Tong Wang et al. "Localized increases in CEPT1 and ATGL elevate plasmalogen phosphatidylcholines in HDLs contributing to atheroprotective lipid profiles in hyperglycemic GCK-MODY", Redox Biology, 2021

33 15 words / < 1% match - Internet from 15-Jul-2020 12:00AM
www.freepatentsonline.com

34 15 words / < 1% match - Internet from 07-Sep-2022 12:00AM
www.hindawi.com

35 15 words / < 1% match - Internet from 02-Mar-2023 12:00AM
www.kjim.org

36 14 words / < 1% match - Crossref
Tsung-Heng Lee, Chih-Ning Cheng, Hsi-Chun Chao, Ching-Hua Lee, Ching-Hua Kuo, Sung-Chun Tang, Jiann-Shing Jeng. "Plasma ceramides are associated with outcomes in acute ischemic stroke patients", Journal of the Formosan Medical Association, 2021

37 14 words / < 1% match - from 02-May-2023 12:00AM
emrc.mui.ac.ir

38 14 words / < 1% match - from 04-Apr-2023 12:00AM
static.frontiersin.org

39 14 words / < 1% match - from 02-Apr-2023 12:00AM
www.humanresourcesonline.net

40 13 words / < 1% match - Crossref
Li, Maoyin, Jonathan E. Markham, and Xuemin Wang. "Overexpression of patatin-related phospholipase AIIIÎ² altered the content and composition of sphingolipids in Arabidopsis", Frontiers in Plant Science, 2014.

41 13 words / < 1% match - Internet from 01-Oct-2019 12:00AM
journals.plos.org

42 13 words / < 1% match - Internet from 31-Aug-2019 12:00AM
www.nature.com

43 12 words / < 1% match - Crossref
"Abstracts 2007", Diabetologia, 2007

44 12 words / < 1% match - Crossref
Leonardo P. de Carvalho, Sock Hwee Tan, Ghim-Siong Ow, Zhiqun Tang et al. "Plasma Ceramides as Prognostic Biomarkers and Their Arterial and Myocardial Tissue Correlates in AcuteMyocardial Infarction", JACC: Basic to Translational Science, 2018

45 12 words / < 1% match - Internet from 23-Oct-2022 12:00AM
academic.oup.com

46 12 words / < 1% match - from 22-Apr-2023 12:00AM
bmcurol.biomedcentral.com

47 12 words / < 1% match - from 28-Jul-2023 12:00AM
cardiab.biomedcentral.com

48 12 words / < 1% match - Internet from 14-Jan-2023 12:00AM
pubs.rsc.org

49 11 words / < 1% match - from 25-May-2023 12:00AM
escholarship.org

50 11 words / < 1% match - Internet from 26-Sep-2022 12:00AM
www.jomes.org

51 10 words / < 1% match - from 27-Jun-2023 12:00AM
Helda.helsinki.fi

52 9 words / < 1% match - Crossref
A. Mantovani, S. Bonapace, G. Lunardi, G. Canali et al. "Associations between specific plasma ceramides and severity of coronary-artery stenosis assessed by coronary angiography", Diabetes & Metabolism, 2019

53	9 words / < 1% match - Crossref<br>Luis Felipe León-Aguilar, Mikael Croyal, Véronique Ferchaud-Roucher, Fengyang Huang et al. "Maternal obesity leads to long-term altered levels of plasma ceramides in the offspring as revealed by a longitudinal lipidomic study in children", International Journal of Obesity, 2018
54	9 words / < 1% match - Crossref<br>Oksana Lavrynenko, Bjoern Titz, Sophie Dijon, Daniel Dos Santos et al. "Ceramide ratios are affected by cigarette smoke but not heat-not-burn or e-vapor aerosols across four independent mouse studies", Life Sciences, 2020
55	9 words / < 1% match - Crossref<br>Tsung-Heng Lee, Chih-Ning Cheng, Chung-Wei Lee, Ching-Hua Kuo, Sung-Chun Tang, Jiann-Shing Jeng. "Investigating sphingolipids as biomarkers for the outcomes of acute ischemic stroke patients receiving endovascular treatment", Journal of the Formosan Medical Association, 2022
56	8 words / < 1% match - Crossref<br>Biyu Hou, Ping He, Peng Ma, Xinyu Yang et al. "Comprehensive Lipidome Profiling of the Kidney in Early-Stage Diabetic Nephropathy", Frontiers in Endocrinology, 2020
57	8 words / < 1% match - Crossref<br>Reijo Laaksonen. "Identifying new Risk Markers and Potential Targets for Coronary Artery Disease: The Value of the Lipidome and Metabolome", Cardiovascular Drugs and Therapy, 2016
58	8 words / < 1% match - Crossref<br>Timon Eckes, Sandra Trautmann, Sonja Djudjaj, Sandra Beyer et al. "Consistent alteration of chain length-specific ceramides in human and mouse fibrotic kidneys", Biochimica et Biophysica Acta (BBA) - Molecular and Cell Biology of Lipids, 2021
59	8 words / < 1% match - Internet from 17-Oct-2010 12:00AM<br>archinte.ama-assn.org
60	8 words / < 1% match - Internet from 12-Nov-2022 12:00AM<br>assets.researchsquare.com
61	8 words / < 1% match - from 15-May-2023 12:00AM<br>warm.dovepress.com
62	7 words / < 1% match - Internet<br>Laaksonen, Reijo, Ekroos, Kim et al. "Plasma ceramides predict cardiovascular death in patients with stable coronary artery disease and acute coronary syndromes beyond LDL-cholesterol.", 'Oxford University Press (OUP)', 2016
63	6 words / < 1% match - Crossref<br>J. R. Bayascas, S. Wullschleger, K. Sakamoto, J. M. Garcia-Martinez et al. "Mutation of the PDK1 PH Domain Inhibits Protein Kinase B/Akt, Leading to Small Size and Insulin Resistance", Molecular and Cellular Biology, 2008

64

6 words / < 1% match - Crossref

Thuy Anh Vu Pham, Thao Thi Phuong Tran. "The interaction among obesity, Type 2 diabetes mellitus, and periodontitis in Vietnamese patients", Clinical and Experimental Dental Research, 2018
