# Supplementary figures and images for: Ceramide d18:1/24:1 as a potential biomarker to differentiate obesity subtypes with unfavorable health outcomes
Source: Lipids Health Dis. 2023 Oct 4;22:166. doi: 10.1186/s12944-023-01921-0 (PMC10548646; doi:10.1186/s12944-023-01921-0)

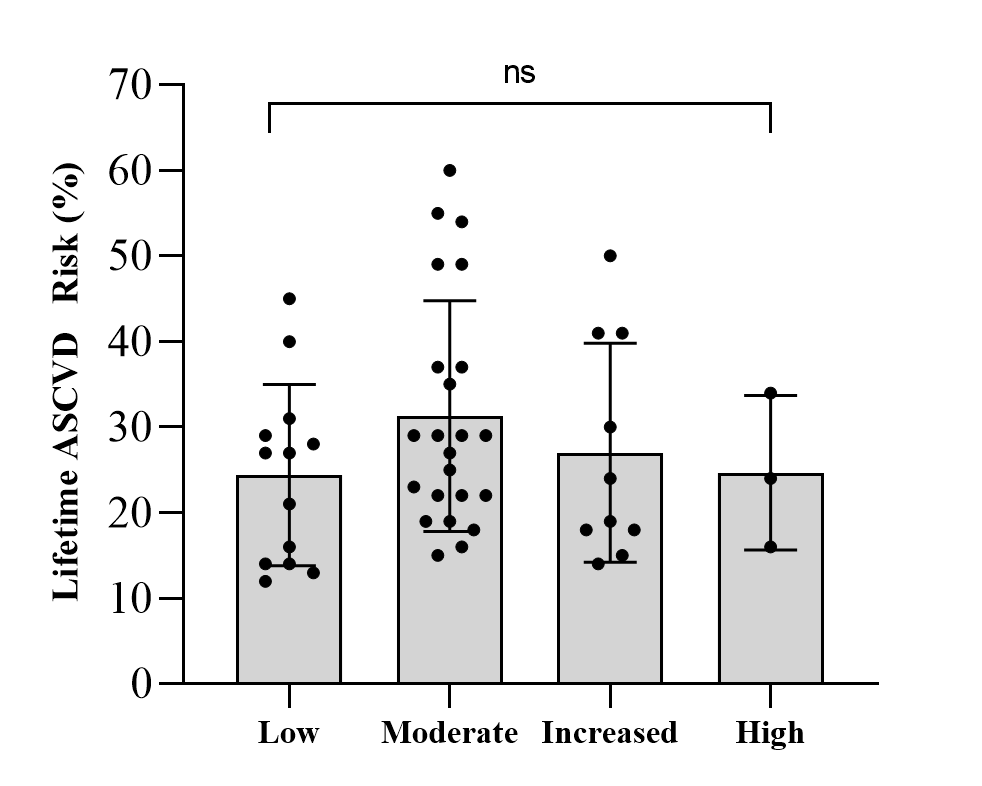

Supplement: Supplementary file 5 — Supplementary Material 5 [file 12944_2023_1921_MOESM5_ESM.tif]
