# Supplementary material for: Ceramide d18:1/24:1 as a potential biomarker to differentiate obesity subtypes with unfavorable health outcomes
Source: Lipids Health Dis. 2023 Oct 4;22:166. doi: 10.1186/s12944-023-01921-0 (PMC10548646; doi:10.1186/s12944-023-01921-0)
Supplement: Supplementary file 6 — Supplementary Material 6 [file 12944_2023_1921_MOESM6_ESM.docx]

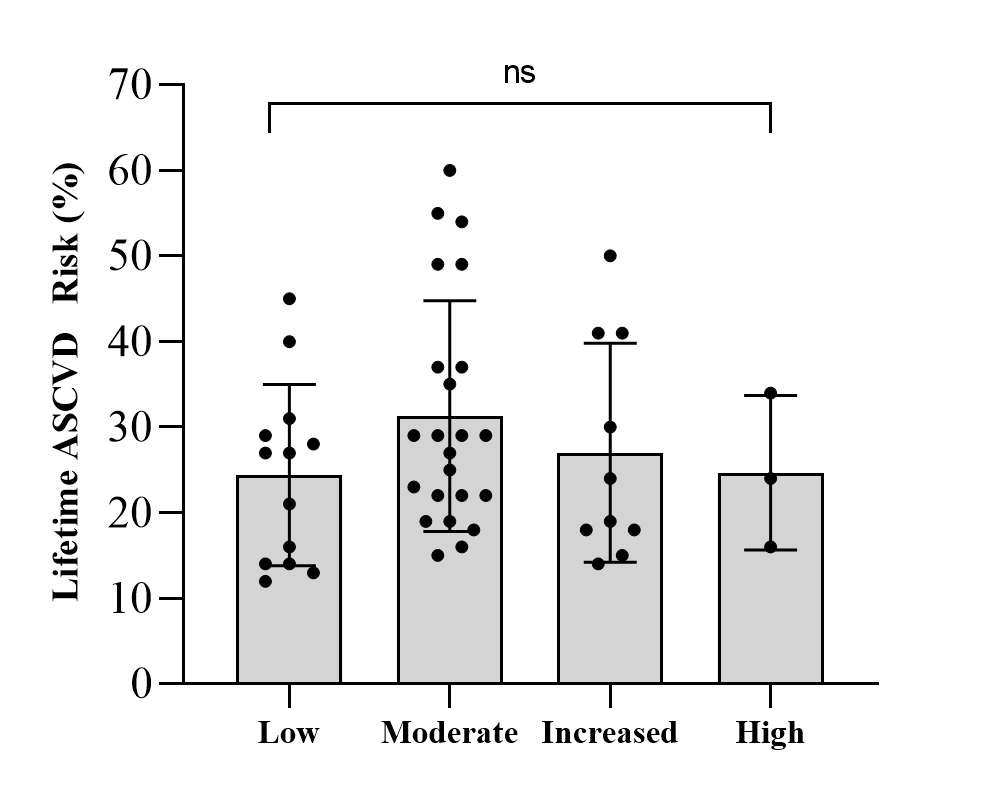
**Supplementary Fig. 1 China-PAR risk score and CERT1 risk score**

49 obese individuals were divided into four groups based on CERT1 risk score (the risks of CVDs are low, moderate, increased and high). The China-PAR risk score of each individual were presented in the figure.
